# Inferring Species Interactions From Co‐occurrence Networks With Environmental DNA Metabarcoding Data in a Coastal Marine Food Web

**DOI:** 10.1111/mec.17701

**Published:** 2025-03-04

**Authors:** Elizabeth Boyse, Kevin P. Robinson, Ian M. Carr, Elena Valsecchi, Maria Beger, Simon J. Goodman

**Affiliations:** ^1^ School of Biology University of Leeds Leeds UK; ^2^ British Antarctic Survey Natural Environment Research Council Cambridge United Kingdom; ^3^ Ceteacean Research and Rescue Unit Banff Aberdeenshire UK; ^4^ Leeds Institute of Medical Research at St James's St James's University Hospital Leeds UK; ^5^ Department of Environmental and Earth Sciences University of Milano‐Bicocca Milan Italy; ^6^ Centre for Biodiversity and Conservation Science, School of the Environment The University of Queensland Queensland Australia

**Keywords:** cetacean, community ecology, co‐occurrence networks, environmental DNA, food webs, species interactions, trophic interactions

## Abstract

A good understanding of biotic interactions is necessary to accurately predict the vulnerability of ecosystems to climate change. Recently, co‐occurrence networks built from environmental DNA (eDNA) metabarcoding data have arisen as a tool to explore interspecific interactions in ecological communities exposed to different human and environmental pressures. Such networks can identify environmentally driven relationships in microbial and eukaryotic communities, but whether inferred co‐occurrences robustly represent biotic interactions remains unclear. Here, we tackle this challenge and compare spatio‐temporal variability in the structure and complexity of inferred co‐occurrence networks and food webs, using 60 eDNA samples covering vertebrates and other eukaryotes in a North Sea coastal ecosystem. We compare topological characteristics and identify highly connected species across spatial and temporal subsets to evaluate variance in community composition and structure. We find consistent trends in topological characteristics across eDNA‐derived co‐occurrence networks and food webs that support some ability for the co‐occurrence networks to detect real ecological processes, despite trophic interactions forming a minority of significant co‐occurrences. The lack of significant trophic interactions detected in co‐occurrence networks may result from ecological complexities, such as generalist predators having flexible interactions or behavioural partitioning, the inability to distinguish age class with eDNA or co‐occurrences being driven by non‐trophic or abiotic interactions. We find support for using eDNA‐derived co‐occurrence networks to infer ecological interactions, but further work is needed to assess their power to reliably detect and differentiate different interaction types and overcome methodological limitations, such as species detection uncertainties, which could influence inferred ecosystem complexity.

## 1 Introduction

Accurately capturing biotic interactions (predation, competition, parasitism, mutualism, commensalism) in ecosystems is challenging given their quantity and complexity (Lee et al. 2019). Understanding these interactions is important as they play a pivotal role in the structure and functioning of ecosystems and in determining species distributions (Gaüzère et al. 2022). Climate change is disrupting species interactions due to contrasting rates of individual species range shifts and altered phenologies, affecting the timing of interactions and potentially accelerating the loss of species and their associated ecosystem functions (Foden et al. 2019; Valiente‐Banuet et al. 2015). Increased knowledge of biotic interactions is crucial to predict the vulnerability of individual species and their ecosystems to environmental perturbations arising from climate change. New tools, such as environmental DNA (eDNA) metabarcoding, can now provide data on community composition across entire ecosystems (Barroso‐Bergada et al. 2021; Bellisario et al. 2021). Species co‐occurrence networks derived from eDNA have been suggested as a way to identify potential species interactions and could support inference of ecosystem responses and vulnerabilities to environmental change (Seymour et al. 2020). However, the power of such networks to differentiate biotic and abiotic interactions and how inferences about community structure can be made from network properties remain poorly explored (Galiana et al. 2024). Here we use eDNA data to construct spatial and temporal co‐occurrence networks for marine communities in coastal waters of the Moray Firth, UK. We then evaluate their ability to distinguish biotic and abiotic contributions to network structure and the implications for inferring ecosystem vulnerability to perturbation.

Interspecific co‐occurrences may be indicative of a biotic interaction whereby two interacting species affect the presence and/or abundance of each other, resulting in non‐random co‐occurrence across space and time (Freilich et al. 2018). Power to detect these interactions in co‐occurrence networks depends on the type and strength of the interaction, and the spatial scale of sampling (Blanchet et al. 2020; Galiana et al. 2024; Morales‐Castilla et al. 2015). The proportion of biotic interactions and which type of interactions contribute to co‐occurrence networks is often uncertain, and the interactions detected can vary greatly among replicate networks, even from the same environment (Barroso‐Bergada et al. 2021; Galiana et al. 2024; Russo et al. 2022). Furthermore, co‐occurrences may also stem from dispersal limitations, response to another species (for example predator avoidance) or abiotic factors, such as shared environmental requirements (Freilich et al. 2018; Galiana et al. 2024), meaning differentiating biotic and abiotic drivers is challenging. To date, eDNA co‐occurrence networks have largely been assembled for microbial, microeukaryotic or meioeukaryotic communities, where limited knowledge of functional roles and biotic interactions prevents validation of the nature of co‐occurrences (Berry and Widder 2014). eDNA co‐occurrence networks derived from other well characterised ecosystems, with known biotic interactions (e.g., trophic interactions derived from diet studies) are necessary to further test their ability to describe biotic interactions.

Trophic interactions are generally better described than other interaction types and have subsequently been used most frequently to identify biotic interactions in co‐occurrence networks (Ford and Roberts 2019). Studies of plankton communities suggest between a quarter and half of co‐occurrences in networks could be trophic in origin, but are constrained by poor taxonomic resolution or the use of presence‐absence rather than quantitative datasets (Freilich et al. 2018; Russo et al. 2023, 2022). Detecting trophic interactions in co‐occurrence networks is particularly challenging as they exhibit strong spatial dependency. Negative co‐occurrences are expected at finer scales where prey are successfully avoiding predators, whereas positive co‐occurrences over greater scales indicate predators tracking their prey (Cazelles et al. 2016; Russo et al. 2023; Thurman et al. 2019). Furthermore, it may be easier to detect trophic interactions between specialist predators and their prey compared with generalist predators as the higher the number of interactions per species, the weaker the interaction strength (Cazelles et al. 2016). Consequently, further exploration comparing known trophic interactions with eDNA co‐occurrences is needed to validate whether trophic interactions are likely to be detected and to determine the spatial influence on these relationships. If eDNA co‐occurrence networks can successfully detect trophic interactions, this could enhance our knowledge of the spatio‐temporal variability of trophic interactions, which is often poorly described relative to overall trophic relationships (Young et al. 2015). This requires a community with well‐known trophic interactions (e.g., from diet‐based food webs), and spatial and temporal structure in predator–prey relationships.

Species interactions in the North Sea ecosystem meet these criteria as they are well characterised from work quantifying impacts from fishing pressure and climate change, presenting a suitable system to further understand the drivers of co‐occurrence networks (Heath 2005; Lynam et al. 2017). The North Sea represents a ‘wasp‐waist’ system, where a few key forage fish species such as sandeels (*Ammodytes* spp.), herring and sprat (clupeids), exert control over the abundance of predators including marine mammals, predatory fishes, and seabirds, through bottom‐up interactions, and influence zooplankton prey through top‐down interactions (Boyse et al. 2024; Fauchald et al. 2011; Lynam et al. 2017; Robinson et al. 2023). Given the importance of these forage fish species and their central role in the North Sea ecosystem, we would therefore expect these species to be highly connected in co‐occurrence networks if trophic interactions are playing a dominant role in forming significant co‐occurrences. Seasonality will likely alter the number of interactions detected between keystone forage fish and their predators or prey in co‐occurrence networks, whereby sandeels are more abundant and therefore targeted as prey more so in early summer (June–July) whilst clupeids are far more abundant from August onwards (Boyse et al. 2024). We would also expect the likelihood of interactions being detected to change depending on diet and foraging specialisations. For example, some seabirds, such as Atlantic puffins ( *Fratercula arctica* ) and black‐legged kittiwakes ( *Rissa tridactyla* ), feed predominantly on forage fish (both sandeels and clupeids) whilst the European shag (*Gulosus aristotelis*) will also target benthic fish species (Wanless et al. 2018). Similarly, central placed foragers, such as seals during the breeding season, are sensitive to local prey depletions as they are limited to foraging within a set distance from breeding and/or resting sites (Engelhard et al. 2014). Forage fish species are vulnerable to climate change because of their reliance on specific substrates (e.g., for burrows or spawning grounds) so co‐occurrence networks could be a useful method to monitor changing interactions in the future, for example, due to reduced temporal synchrony between predators and prey during seasonal foraging periods, if trophic interactions are well characterised within these networks (Frederiksen et al. 2011; Petitgas et al. 2013).

Here, we aim to evaluate how eDNA‐derived co‐occurrence network properties reflect ecological interactions when interpreting key ecosystem components and their potential to support inferences about vulnerability to change. We use eDNA‐derived occurrence data for marine eukaryotes from a well‐studied ecosystem, the Moray Firth, an embayment within the North Sea. Specifically, our objectives are to match known trophic interactions to co‐occurrence networks to (1) quantify the number of trophic interactions detected, (2) understand whether these interactions are more likely to be negative or positive and (3) evaluate whether specialist predators have more interactions than generalist predators. We expect high numbers of trophic interactions will be matched to co‐occurrence links involving key forage fish species, sandeels and clupeids, given their central role in North Sea food webs (Stäbler et al. 2018). Second, trophic interactions require actual co‐occurrence of two interacting species, but predator avoidance strategies could present as negative interactions (species systematically do not co‐occur). Commonly occurring marine mammals in the North Sea have well‐defined specialised diets, so we assume trophic interactions between these species and their dominant prey species to be detected in co‐occurrence networks (Boyse et al. 2024; Robinson et al. 2023). Overall, we expect that nearshore and early‐season community networks will be more complex, with higher numbers of species interactions due to higher species diversity (Boyse et al. 2024).

## 2 Materials and Methods

### 2.1 Sample Collection and Analysis

We employed an eDNA metabarcoding dataset derived from 60 10 L seawater samples collected on four sampling trips during June to October 2021 to assess spatial and temporal community changes from the southern Moray Firth (Boyse et al. 2024). Seawater samples were filtered within 6 days of collection, 89% within 3 days of collection, and then stored at −20°C until DNA extractions were carried out with the Qiagen DNeasy PowerSoil Pro Kit. Marine vertebrate DNA was amplified using two primer sets, MarVer1 and MarVer3, targeting 12S and 16S rRNA barcode markers, respectively (Valsecchi et al. 2021, 2020), as well as eukaryotic DNA with 1391F and EukBr, targeting the V9 region of 18S rRNA, to capture zooplankton and other invertebrate taxa (Amaral‐Zettler et al. 2009; Sawaya et al. 2019). Sequencing libraries were prepared and sequenced separately at the University of Leeds Genomics Facility, St James Hospital, using an Illumina MiSeq Sequencer with a 150‐bp paired‐end lane for each of the vertebrate primer sets and a 250 bp paired‐end lane for 18S rRNA (Boyse et al. 2024). The bioinformatics pipeline is described fully in Valsecchi et al. (2020) and can be found at http://www.dna‐leeds.co.uk/eDNA/. Following the removal of low‐quality sequences, PCR duplicates and chimaeras, we clustered sequences into molecular operational taxonomic units (OTUs) using a 98% threshold of homology to the GenBank sequence at the species level for the two vertebrate primers and a 95% threshold at the class level for the 18S primer set (Bonin et al. 2023). We reviewed all cluster assignments manually to validate taxonomic identifications. Read counts were converted into an OTU‐specific index, allowing comparison of within‐OTU abundances across space and time between different primer sets and samples based on the assumption that amplification efficiency is constant for a given taxon and primer set (Djurhuus et al. 2020; Kelly et al. 2019). Firstly, we converted read counts into proportions, then divided the maximum proportion for each OTU from the proportion at individual sites, resulting in an index between 0 and 1 for each OTU (Kelly et al. 2019). For vertebrate OTUs that were present across both primer sets, we built an ensemble OTU index by taking the average across both indices at each site (Djurhuus et al. 2020).

### 2.2 Co‐occurrence Network Construction

We subset our dataset into groups to account for spatial and temporal trends in community composition for co‐occurrence analyses based on non‐metric multidimensional scaling (NMDS) and permutational multivariate analysis of variance (PERMANOVA) (Boyse et al. 2024), as more similar communities produce more specific co‐occurrence networks (Berry and Widder 2014). We will refer to early season, June to July (34 sites), and late season subsets, August to October (23 sites), as ‘temporal subsets’ and will retain spatial signals in the dataset. Nearshore (13 sites; < 1.2 km from shore) and offshore (47 sites; 2.5–16 km offshore) subsets will be called ‘spatial subsets’ and preserve temporal patterns in the dataset. Small sample sizes (< 20) can affect the reliability of co‐occurrence networks to accurately predict interactions, so extra caution must be applied to networks produced with sample numbers below this threshold (Hirano and Takemoto 2019). For each subset, we only retained OTUs that appeared in at least 25% of the sites, thereby removing rare species and reducing erroneous correlations in our dataset (Berry and Widder 2014).

We assembled individual co‐occurrence networks by calculating pairwise co‐occurrences between species OTU indexes, a measure of relative abundance, with five different metrics (Pearson and Spearman correlations, Bray–Curtis and Kullback–Leibler dissimilarities, and mutual information) in Cytoscape's Conet plugin (Faust and Raes 2016). Edges, that is, significant co‐occurrences between two species, were represented in the network if they were supported by at least two metrics, reducing the likelihood of false positives, with the highest and lowest scoring 500 edges being retained to capture both positive and negative interactions (Faust and Raes 2016). *P* values were calculated using the ReBoot method which compares the null distribution of correlations, accounting for compositionality, using 100 iterations of method and edge specific renormalised permutations, and 100 iterations of bootstrapped confidence intervals of observed correlations (Faust et al. 2012). *P* values across different metrics were then merged using Brown's method and corrected for multiple testing with the Benjamini–Hochberg approach (Benjamini and Hochberg 1995; Brown 1975).

### 2.3 Food Web Construction

We used a meta‐web approach to construct food webs for the Moray Firth ecosystem by determining all potential trophic interactions between consumers and resources detected by our eDNA metabarcoding dataset (D'Alessandro and Mariani 2021). We downloaded diet items for fishes, marine mammals and seabirds from FishBase (https://www.fishbase.se; accessed 14/12/2022) and SeaLifeBase (https://www.sealifebase.ca; accessed 6/12/2022), through the ‘rfishbase’ R package version 4.0.0 (Boettiger et al. 2012). We complemented these data with information from the primary literature, including invertebrate species, using the Google Scholar search engine and search terms ‘Latin species name’ or ‘common species name’ with ‘feeding’, ‘diet’ or ‘stomach contents’. For some well‐studied species, such as marine mammals or seabirds, we restricted the search to dietary studies within the North Sea. We constructed an edge list describing all possible consumer–resource interactions, and subset the data as described above for co‐occurrence networks, removing rare species that were present in less than 25% of samples.

Topological properties for both co‐occurrence networks and food webs were subsequently calculated using Cytoscape NetworkAnalyzer (Assenov et al. 2008). Food webs and networks were visualised with the iGraph R package version 1.2.1 (Csardi and Nepusz 2006). Trophic levels for vertebrate species were assigned from FishBase and SeaLifeBase records. We assigned primary producers (i.e., algae) and fungi to trophic level 1, and all other invertebrate classes to trophic level 2 for the purpose of this study. Significant co‐occurrences that represented trophic interactions were inferred from the literature used to build food webs. We identified potential keystone species as those with the highest degree of co‐occurrences or interactions from co‐occurrence networks and food webs, respectively (Berry and Widder 2014).

## 3 Results

### 3.1 Moray Firth Community Composition

We retrieved 6,894,772 sequences assigned to 88 OTUs across both vertebrate primer sets, and 1,469,355 sequences assigned to 36 OTUs for 18S rRNA (Boyse et al. 2024). Over 90% of vertebrate reads belonged to teleost fishes, although mammals, Chondrichthyes and birds were also detected. OTUs with the most abundant read counts included forage fish, such as sandeels, clupeids and mackerel ( *Scomber scombrus* ). Classes from Animalia (48% total reads) and Chromista (41% total reads) contributed relatively equally to overall eukaryotic reads. Copepods from the Maxillopoda Class comprised most of the Animalia reads, whereas dinoflagellates (Dinophyceae) and diatoms (Bacillariophyceae) both dominated Chromista read counts (Boyse et al. 2024). There was a clear temporal signal in community composition, differing significantly between early and late season samples for vertebrates, and across all four sampling months for eukaryotes (Boyse et al. 2024). Sandeels were more prevalent in the early season, whereas mackerel were more abundant in the late season. Maxillopoda accounted for most reads in the first and last sampling months, whereas Dinophyceae were more prevalent in the middle sampling months (July and August). For both vertebrates and broader eukaryotes, the nearshore community (< 1000 m from shore) had higher alpha diversity and significantly different beta diversity from communities composed of samples collected further offshore (Boyse et al. 2024). Numerous fish species and eukaryote classes were found exclusively in the nearshore community that are known to be associated with shallow depths.

### 3.2 Temporal Food Webs and Co‐occurrence Network Subsets

**FIGURE 1:**
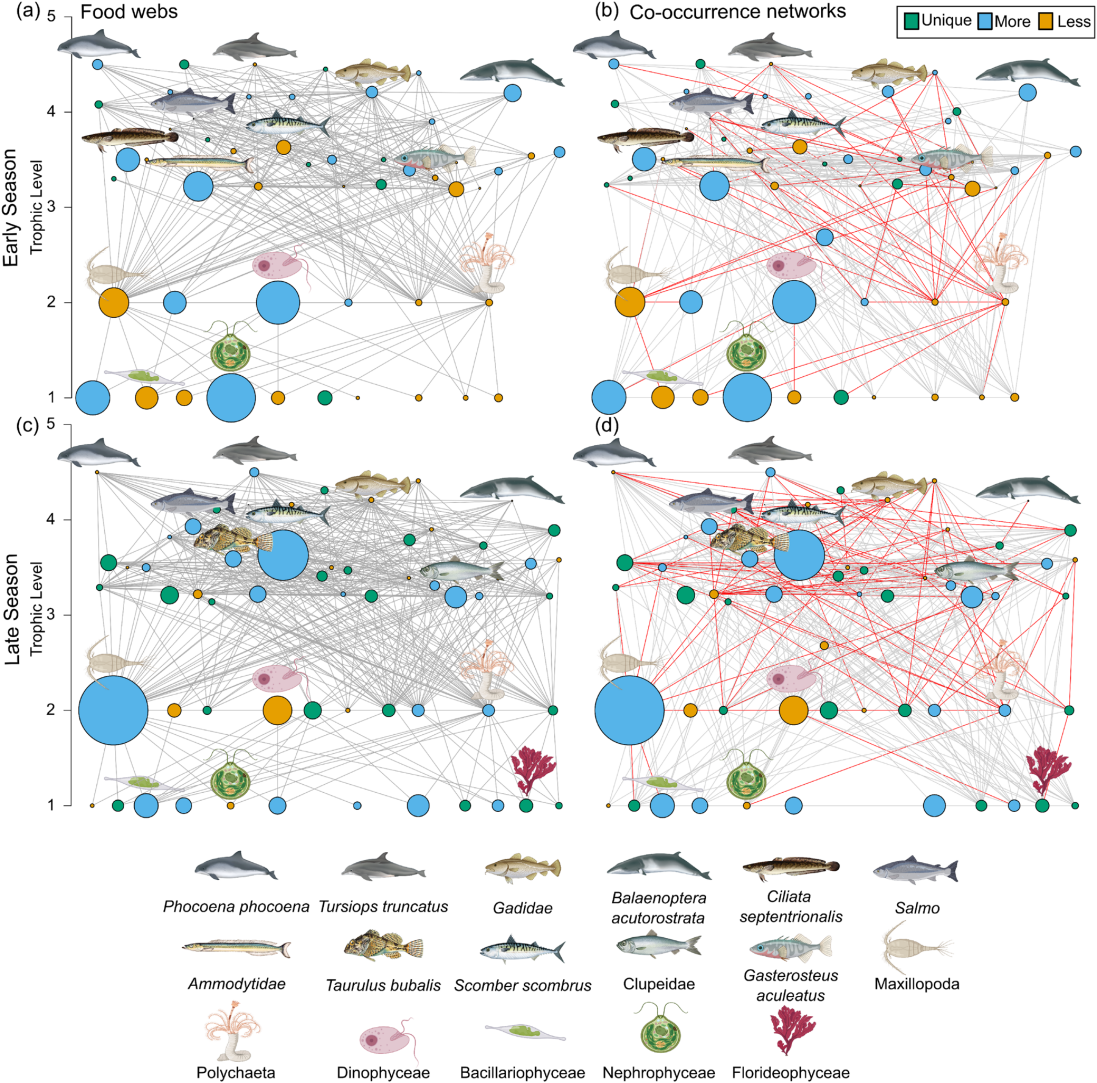
Food webs from (a) early season (June to July) and (c) late season (August to October), determined by environmental DNA metabarcoding detections and known trophic interactions. Respective co‐occurrence networks, built with pairwise co‐occurrences using 5 different metrics (correlations, dissimilarities, mutual information) and eDNA relative abundance data, for the (b) early season and (d) late season. The size of the node represents the scaled average abundance of the molecular operational taxonomic unit (OTU) across samples, and the colour indicates whether the OTU is unique to that time period (green) or more (blue) or less (yellow) abundant. Red edges in co‐occurrence networks signify realised trophic interactions on the basis of the putative food webs, whereas grey edges represent all other significant co‐occurrences. Individual OTUs are plotted in the same location between graphs.

Early season (June and July) co‐occurrence networks and food webs had six and nine fewer nodes, and 108 and 172 fewer edges, respectively, compared with the late season (August to October) (Table 1). Edges in the co‐occurrence networks were dominated by positive interactions, representing 85.6% of edges in the early season and 77.9% in the late season, resulting from OTU co‐presences (Figures S1 and S2). Only 53 (17%) edges and 104 (25%) edges in the co‐occurrence networks represented realised trophic interactions on the basis of our putative food webs. Potential keystone OTUs, that is, highly connected OTUs, did not overlap between temporal subsets for the co‐occurrence networks and food webs, apart from sandeels, which were a potential keystone OTU for the late season co‐occurrence network and both food web subsets (Table 2). Alternative metrics for identifying potential keystone OTUs, closeness centrality and betweenness centrality, highlighted similar species (Tables S1 and S2). We found a negative correlation between average OTU abundance and degree for the early season co‐occurrence network (Pearson, *r* = −0.41, *p* < 0.05), but no pattern for the late season network. For example, sandeels only showed a high degree of edge interactions in the late season when they were less abundant (Figure 1). OTUs with the most edge interactions in food webs were dominated by species that occupied mid trophic levels (2–3), as both consumers and prey within the ecosystem (Figure 1). This included some of the most abundant OTUs detected, such as copepods, mackerel, sandeels and clupeids.

**TABLE 1:** Topological characteristics of temporal co‐occurrence networks (undirected) and food webs (directed) for early season (June to July) and late season (August to October) Moray Firth subsets.

	Co‐occurrence networks	Food webs		
	Early season	Late season	Early season	Late season
Nodes^a^	54	60	53	62
Edges^b^	305	413	290	462
Avg. neighbours^c^	11.3	13.767	10.49	14.52
Diameter^d^	4	4	5	6
Radius^e^	3	2	1	1
Characteristic path length^f^	2.03	1.94	1.87	1.88
Clustering coefficient^g^	0.43	0.44	0.34	0.34
Network density^h^	0.21	0.23	0.11	0.12

^a^ Nodes represent OTUS.

^b^ Edges represent interspecific co‐occurrences in co‐occurrence networks, or trophic interactions in food webs.

^c^ Average number of neighbours refers to the average number of edges per node.

^d^ The diameter is the maximum length of the shortest path between two nodes.

^e^ The radius is the minimum length of the shortest path between two nodes.

^f^ The characteristic path length is the average shortest path length between any two nodes in the network.

^g^ The clustering coefficient represents the average clustering coefficient across all nodes and is a value between 0 and 1. It represents the proportion of co‐occurrences among the neighbours of a node.

^h^ Network density describes the proportion of realised co‐occurrences from all potential co‐occurrences.

**TABLE 2:** Potential keystone species, identified as the 10 molecular operational taxonomic units (OTUs) with the highest degree (number of edges), identified in co‐occurrence networks and food webs from the early season (June to July) and late season (August to October) Moray Firth subsets. The colour of the box indicates the trophic level of each OTU, from white (trophic level 1) to black (trophic level 4).

Co‐occurrence networks		Food webs	
Early season	Late season	Early season	Late season
Phaeophyceae	*Oncorhynchus*	Polychaeta	Polychaeta
*Spinachia spinachia*	Dinophyceae	Gadidae	Gadidae
*Dicentrarchus*	*Taurulus bubalis*	Maxillopoda	Maxillopoda
*Symphodus melops*	Ammodytidae	Clupeidae	Bivalvia
*Centrolabrus exoletus*	*Gasterosteus aculeatus*	Gastropoda	*Scomber scombrus*
*Gasterosteus aculeatus*	*Zoarces*	Ammodytidae	Clupeidae
*Taurulus bubalis*	*Larus argentatus*	*Scomber scombrus*	Gastropoda
*Pholis gunnellus*	*Anguilla anguilla*	*Pomatoschistus minutus*	Ammodytidae
*Chirolophis ascanii*	*Pholis gunnellus*	Pleuronectidae	Pleuronectidae
*Salmo*	*Phocoena phocoena*	*Trisopterus esmarkii*	*Pomatoschistus minutus*

### 3.3 Spatial Food Webs and Co‐occurrence Network Subsets

**FIGURE 2:**
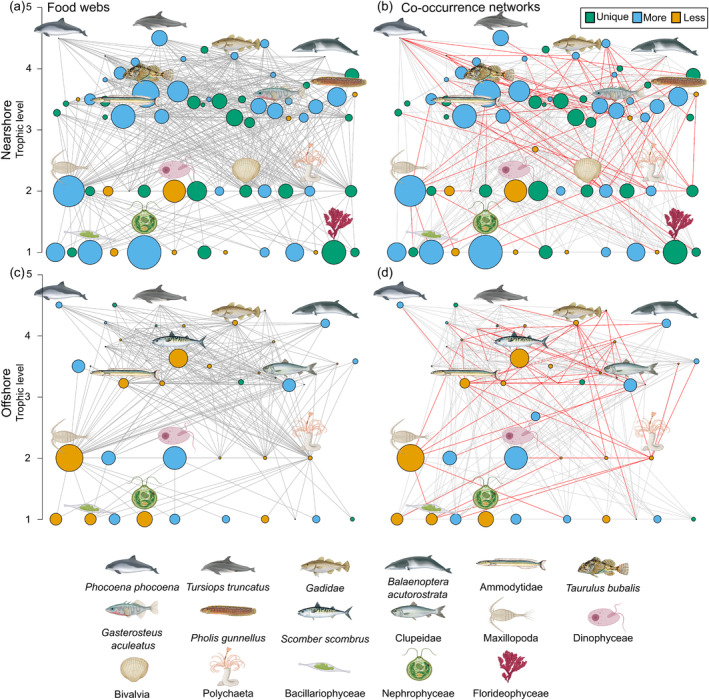
Food webs from the (a) nearshore community with samples collected < 1000 m from shore, and (c) offshore community determined by environmental DNA metabarcoding detections and known trophic interactions. Respective co‐occurrence networks, built with pairwise co‐occurrences using 5 different metrics (correlations, dissimilarities and mutual information) and eDNA relative abundance data, for the (b) nearshore and (d) offshore communities. The size of the node represents the scaled average abundance of the molecular operational taxonomic unit (OTU) across samples, and the colour indicates whether the OTU is unique to that time period (green) or more (blue) or less (yellow) abundant. Red edges in co‐occurrence networks signify realised trophic interactions on the basis of the putative food webs, whereas grey edges represent all other significant co‐occurrences. Individual OTUs are plotted in the same location between graphs.

We detected 22 more OTUs in the nearshore community compared with offshore, despite the nearshore community, including only 13 samples compared with 47 offshore samples (Table 3). This included 27 OTUs that were only found in the nearshore community (Figure 2). More edges were formed between nodes for the nearshore community, with 139 more edges for the co‐occurrence networks and 206 more edges for the food webs. The spatial subsets also detected a greater proportion of co‐presences (nearshore 73.6% and offshore 77.9%), compared with mutual exclusions (Figures S3 and S4), and a small proportion of trophic interactions (nearshore 20.9%, offshore 22.5%). Similar to the temporal subsets above, we discovered little overlap between potential keystone OTUs in the co‐occurrence networks and food webs, with Gastropoda being the only potential keystone OTU in both the nearshore co‐occurrence networks and food webs, and sandeels the only overlapping keystone OTU offshore (Table 4). Two keystone OTUs, the three‐spined stickleback ( *Gasterosteus aculeatus* ) and the long‐spined bullhead ( *Taurulus bubalis* ), were shared across all four co‐occurrence networks. Conversely, five keystone OTUs were shared by one spatial subset and one temporal subset: dinoflagellates (Dinophyceae), brown algae (Phaephyceae), harbour porpoise ( *Phocoena phocoena* ) and salmon/trout genera (*Salmo*, *Oncorhynchus*). We found a positive correlation between average OTU abundance and degree for the nearshore network (Pearson, *r* = 0.31, *p* < 0.05), but no correlation was detected for the offshore network.

**TABLE 3:** Topological characteristics of spatial co‐occurrence networks (undirected) and food webs (directed) for nearshore (< 1000 km) and offshore communities.

	Co‐occurrence networks	Food webs		
	Nearshore	Offshore	Nearshore	Offshore
Nodes^a^	68	46	67	46
Edges^b^	383	244	465	259
Avg. neighbours^c^	11.27	10.61	13.55	10.826
Diameter^d^	4	3	5	4
Radius^e^	3	2	1	1
Characteristic path length^f^	2.13	1.88	1.86	1.8
Clustering coefficient^g^	0.38	0.4	0.31	0.36
Network density^h^	0.17	0.24	0.11	0.13

^a^ Nodes represent OTUS.

^b^ Edges represent interspecific co‐occurrences in co‐occurrence networks, or trophic interactions in food webs.

^c^ Average number of neighbours refers to the average number of edges per node.

^d^ The diameter is the maximum length of the shortest path between two nodes.

^e^ The radius is the minimum length of the shortest path between two nodes.

^f^ The characteristic path length is the average shortest path length between any two nodes in the network.

^g^ The clustering coefficient represents the average clustering coefficient across all nodes and is a value between 0 and 1. It represents the proportion of co‐occurrences among the neighbours of a node.

^h^ Network density describes the proportion of realised co‐occurrences from all potential co‐occurrences.

**TABLE 4:** Potential keystone operational taxonomic units (OTUs), identified as the 10 molecular OTUs with the highest degree (number of edges), from co‐occurrence networks and food webs from the nearshore and offshore communities. The colour indicates the trophic level of each OTU, from white (trophic level 1) to black(trophic level 4).

Co‐occurrence networks		Food webs	
Nearshore	Offshore	Nearshore	Offshore
*Zoarces*	Dinophyceae	Polychaeta	Polychaeta
Calcarea	*Taurulus bubalis*	Maxillopoda	Gadidae
Ascidiacea	*Limanda limanda*	Gadidae	Maxillopoda
Granuloreticulosea	Ammodytidae	Bivalvia	Clupeidae
*Taurulus bubalis*	*Salmo*	Gastropoda	Ammodytidae
*Phocoena phocoena*	*Gasterosteus aculeatus*	*Scomber scombrus*	*Scomber scombrus*
Phaeophyceae	*Oncorhynchus*	Clupeidae	Gastropoda
*Gasterosteus aculeatus*	*Tursiops truncatus*	Pleuronectidae	*Pomatoschistus minutus*
Gastropoda	Bacillariophyceae	Ammodytidae	Pleuronectidae
Chlorodendrophyceae	*Ctenolabrus rupestris*	Ophiuroidea	*Trisopterus esmarkii*

### 3.4 Stability of Edges Between Different Co‐occurrence Network Subsets

**FIGURE 3:**
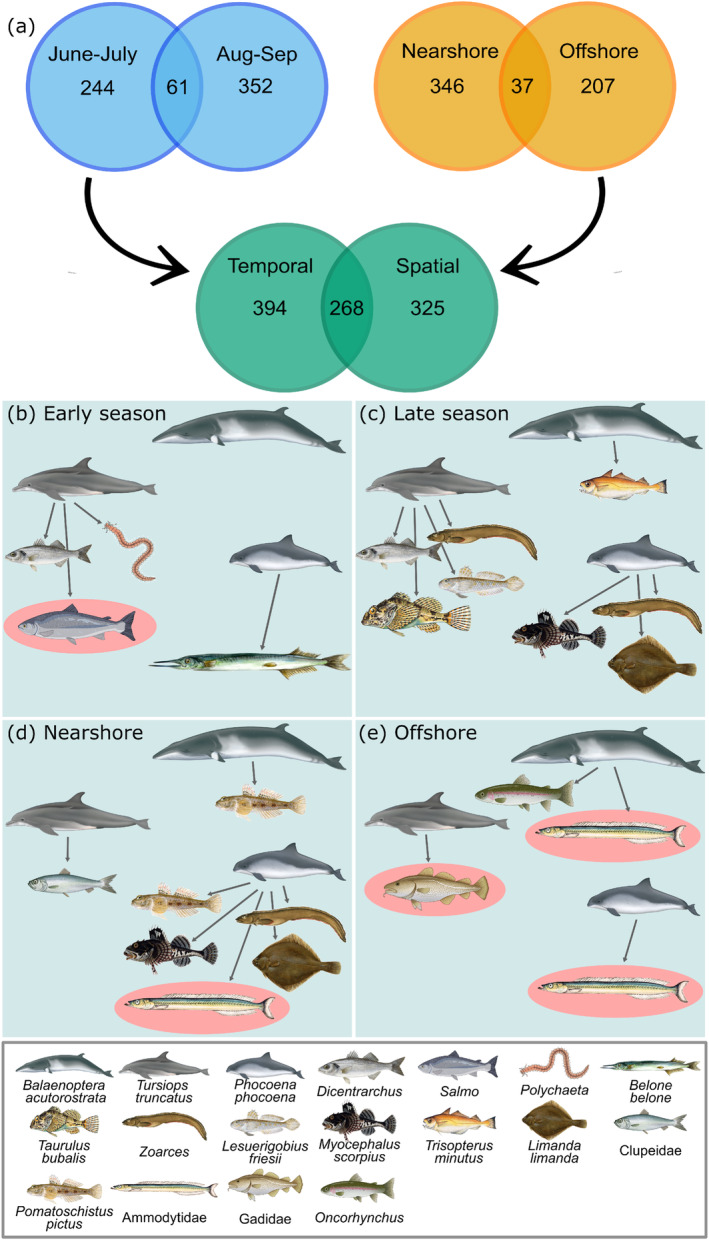
(a) Venn diagrams showing the number of overlapping edges detected in the different co‐occurrence network subsets. ‘Spatial’ represents all edges in the monthly samples combined and ‘Temporal’ represents all edges in the nearshore and offshore communities combined, with duplicates removed. (b) to (e) show trophic interactions between cetaceans and prey in temporal (early season and late season) and spatial (nearshore and offshore) co‐occurrence network subsets. Red ellipses indicate known dominant prey species.

Within the temporal and spatial co‐occurrence network subsets, there were a high number of unique edges, with only 61 (9%) and 37 (6%) shared edges in the temporal and spatial subsets, respectively (Figure 3a). Only 268 edges (27%) were found in both subsets, with a similar number of edges unique to the temporal or spatial subsets, 394 and 325 edges, respectively. We investigated edge stability further with cetacean trophic interactions and found very few overlapping edges between subsets (Figure 3b). Only two trophic interactions between bottlenose dolphins ( *Tursiops truncatus* ) and European seabass (*Dicentrachus*), and between harbour porpoises and sandeels were detected in more than one subset. Only 21% of the detected trophic interactions were with known dominant prey species, and these interactions were all unique to one subset, apart from those between harbour porpoises and sandeels which were detected in both nearshore and offshore networks.

## 4 Discussion

Less than a quarter of significant co‐occurrences were attributable to trophic interactions. Consequently, dominant forage fish species were not as highly connected in co‐occurrence networks compared with food webs and appeared to have higher co‐occurrence linkages in networks where they are known to be less abundant and not preyed upon, such as sandeels in the late season when they are residing in sandy burrows. Despite common marine mammals in the North Sea being specialist predators, often targeting a few key prey species, these did not always appear as co‐occurrence linkages in the networks. Instead, interactions contributing to different co‐occurrence networks were highly changeable, with only 27% of interactions shared between the spatial and temporal subsets despite the data being the same, highlighting the importance of methodological decisions in the resulting interactions detected.

Topological characteristics did not vary greatly between co‐occurrence networks and food webs, or between spatial and temporal subsets (Tables 1 and 3), which is expected given the small spatial scale (tens of kilometres) of sampling in the present study. The nearshore network was produced from fewer samples (13 samples) but shared similar topological characteristics with the other networks, suggesting the small sample size had a limited impact on retrieving significant interactions (Hirano and Takemoto 2019). The most notable differences were found in the number of edges, with higher interaction diversity detected in the late season and nearshore networks. Networks with more interactions could indicate higher ecosystem functionality and greater redundancy of interactions, thus increasing the community's resilience to disturbance (Tylianakis et al. 2010; Valiente‐Banuet et al. 2015). Higher interaction richness in the nearshore subset was likely driven by greater species richness, which has been found to increase closer to shore in previous eDNA metabarcoding studies (Jiménez et al. 2018; O'Donnell et al. 2017; Ríos Castro et al. 2022).

Despite the widespread application of eDNA metabarcoding data for building co‐occurrence networks of microorganisms, methodological limitations impacting topological characteristics have not been addressed sufficiently in these studies to date. For example, the higher species richness of the nearshore environment could be a methodological artefact, as the water samples were collected from surface water (at 4 m depth; Boyse et al. 2024), such that more benthic species might be detected in the shallower nearshore samples. Previous studies employing eDNA metabarcoding have detected benthic species at deeper depths (> 200 m) because of the presence of eddies (O'Donnell et al. 2017), although stratification in the southern Moray firth may prevent DNA mixing in the water column (Adams and Martin 1986). Notably, our 18S primer set targeting eukaryotes failed to amplify DNA from organisms in the Malacostraca class (Crustacea), such as amphipods, carideans, decapods and isopods. We recommend using COI and 18S in combination for future North Sea zooplankton metabarcoding studies to provide a more comprehensive overview of community composition, and allow higher taxonomic resolution (Lacoursière‐Roussel et al. 2018). Although copepods dominate North Sea plankton assemblages and are the major component of forage fishes' diets, these other crustacean groups still form part of the diet of most planktivorous forage fish, and therefore would be reasonably well connected in food webs, potentially becoming more important as copepod abundance declines with warming waters (Garzke et al. 2015; Mortelmans et al. 2021; Segers et al. 2007). Furthermore, other potentially important OTUs may have been removed because of incomplete reference libraries, affecting the overall ecosystem complexity, as coverage of North Sea macrofauna with the 18S gene is only approximately 36.4% to date (Sawaya et al. 2019; Zamkovaya et al. 2021). Extracting abundance data from eDNA metabarcoding is debated, especially in relation to methods for tackling bias stemming from differing amplification efficiencies (Hansen et al. 2018; Hestetun et al. 2020; Shelton et al. 2023). Here, we transformed our read counts into an OTU‐specific index, which assumes the amplification efficiency is constant across all samples regardless of the community composition (Kelly et al. 2019). However, species composition will likely affect the read numbers retrieved, with more accurate abundance estimates recovered for dominant taxa compared with rarer taxa (Skelton et al. 2023). All these effects could potentially impact interaction diversity and network complexity, so must therefore be acknowledged as shortfalls when pairing eDNA metabarcoding data with co‐occurrence network analyses.

Only a small proportion of significant co‐occurrences were attributable to trophic interactions in the present analyses. Our food webs showed that most predators within the study area feed on multiple prey species (Figures 1 and 2), thus reducing the likelihood of detecting trophic interactions due to dietary plasticity (Robinson et al. 2023; Thurman et al. 2019). Some trophic interactions may not be described in the literature and are subsequently categorised as non‐trophic interactions in our analyses. For example, dinoflagellates, which often make up a significant component of plankton biomass and are both important consumers and a food resource, were not well connected in our food webs, likely owing to difficulties identifying planktonic species in stomach content analyses (Pethybridge et al. 2014; Sherr and Sherr 2007). In this case, co‐occurrence network analyses can potentially contribute to our current understanding, as dinoflagellates are an abundant and well‐connected OTU in the offshore network, suggesting they may play an important role in this community. Increased diet metabarcoding studies will thus serve to improve our knowledge of trophic interactions in plankton communities in the future (Zamora‐Terol et al. 2020).

Detecting trophic relationships in co‐occurrence networks relies on the assumption that predators are tracking their prey (co‐presence) or prey are avoiding their predators (mutual exclusion) (Thurman et al. 2019). In reality, however, species will partition their time between different behaviours or, at the extreme end of the scale, show seasonality in their foraging and breeding grounds, which will affect interspecific interactions over different spatio‐temporal scales (Risch et al. 2014). An important limitation to eDNA sampling, which is often overlooked in co‐occurrence networks, is that the method is unable to distinguish between age classes, for example, larval versus adult stages (Hansen et al. 2018). Age substantially influences what a species eats and who it is eaten by (Bossier et al. 2020). For example, herring will consume juvenile cod, but adult cod will also consume herring, which may skew correlation trends when age class cannot be distinguished (Lynam et al. 2017). Spawning events may additionally result in increased peaks in eDNA abundances, which could further bias interactions towards those incorporating early life stages, as the presence of DNA derived from gametes may not reflect the occurrence of trophic interactions in the same way as for older age classes (Di Muri et al. 2023; Valsecchi et al. 2021). New approaches utilising environmental RNA to estimate species ages may be incorporated into eDNA‐based networks in the future to alleviate this problem (Barratclough et al. 2024; Stevens and Parsley 2023).

We discovered high numbers of edges unique to either the spatial or temporal subsets, despite the same data being used to create these independent subsets (Figure 3). Designating subsets prior to co‐occurrence analyses is often carried out arbitrarily, but samples must come from similar environments to avoid habitat filtering dominating potential interactions (Berry and Widder 2014). In this study, we focused on trophic interactions with cetacean species to investigate edge stability, as we know that cetacean diets are typically dominated by a few target prey species, thus increasing the likelihood of detecting the trophic interactions present (Thurman et al. 2019). For example, sandeels and clupeids comprise over 80% by weight of minke whale ( *Balaenoptera acutorostrata* ) diets in Scottish waters, whereas sandeels and whiting (Gadidae) make up 80% of harbour porpoise diets (Pierce et al. 2004; Santos et al. 2004). However, significant co‐occurrences between sandeels and minke whales or harbour porpoises were only detected in the spatial co‐occurrence subsets (Figure 3). This may be due to the spatial subsets retaining a temporal signal, and the abundance of sandeels displays strong temporal variability with higher abundances in June and July when they are actively feeding within the water column (Henriksen et al. 2021; Boyse et al. 2024). Similarly, we also detected higher abundances of both minke whales and harbour porpoises in June and July. Gadidae species contribute up to 84% of the bottlenose dolphin diet, and salmon are also suspected to be a dominant prey species, although their otoliths are almost completely digested so are inherently difficult to detect in stomach content analyses (Santos et al. 2001). We found co‐occurrences between bottlenose dolphins and Gadidae in the offshore network, and with salmon in the early season network, when species occurred at very low abundances. Zurell et al. (2018) previously observed that predator–prey co‐occurrences were more likely to be detected when both species were rare, and detectability decreased as one of the species became more abundant. However, rare species in eDNA analyses may represent false positive detections stemming from the transport of DNA in tides and currents (Hansen et al. 2018). All three of these cetacean species also displayed trophic interactions with species not known to form large portions of their diet, which could indicate prey switching, although this seems unlikely as their targeted prey were available in higher relative abundances.

Potential keystone OTUs in co‐occurrence networks and food webs were found to be largely different (Tables 2 and 4). We identified potential keystones on the basis of degree centrality, but OTUs with high closeness and betweenness centralities were largely very similar (Appendices A3.1 and A3.2). The three‐spined stickleback and long‐spined bullhead were the only two OTUs that exhibited high degree centrality across all four co‐occurrence networks (Tables 2 and 4). The functional role of these species in ecosystems is not well understood, especially non‐trophic interactions which make up the majority of interactions within our networks. However, both OTUs were most abundant in the nearshore environment and co‐occurred within all networks, suggesting their co‐occurrence could be driven by similar habitat requirements. The three‐spined stickleback spawns in very shallow coastal environments (< 3 m depth) during the spring and summer but spends most of its life cycle in open seas, whilst the long‐spined bullhead is a permanent resident of the intertidal zone (Bergström et al. 2015; Barrett et al. 2018). These species also share many edges with other predominantly coastal inhabitants, such as the European herring gull ( *Larus argentatus* ), corkwing wrasse ( *Symphodus melops* ), rock gunnel ( *Pholis gunnellus* ), salmon/trout (*Salmo*) and many benthic invertebrate species (bivalves, gastropods, polychaetes) (Figure 2), enhancing the likelihood that co‐occurrences resulted from shared habitat requirements. In this instance, these species are therefore unlikely to be keystone species with disproportionate negative effects across the ecosystem if removed (Faust et al. 2012). The detections of *Oncorhynchus* as a potential keystone species in both the late season and offshore networks most likely represent the invasive pink salmon ( *Oncorhynchus gorbuscha* ) (Boyse et al. 2024; Skóra et al. 2023). In the offshore network, the native *Salmo* was also a potential keystone species, and both salmonids significantly co‐occurred suggesting potential competition between the two species, which has generated concern in recent years as *Salmo salar stocks* are currently declining (Skóra et al. 2023). However, they only shared three co‐occurrences with other species in the network, of which only one, *Gasterosteus aculeatus* , represented a trophic interaction whilst they both had three other trophic interactions that were not shared with each other.

Other OTUs, such as bottlenose dolphins in the offshore network, and sandeels in the late season network, only have high degree centrality in the networks where they are least abundant. We sighted no bottlenose dolphins offshore whilst collecting samples, and previous research corroborates that they are regionally coastal, found in depths of < 25 m and typically no further than 1.5 km from the shore (Culloch and Robinson 2008; Robinson et al. 2007). Therefore, it is likely that the small quantities of eDNA detected for bottlenose dolphins in the offshore environment resulted from the movement of DNA particles in the water column as opposed to the bottlenose dolphins actually being present (Andruszkiewicz et al. 2019). Similarly, sandeels were only detected as a keystone OTU in the late season network when they are known to occur in low abundance and are less available to interact with other species in the water column as they are primarily buried in the sand (Henriksen et al. 2021). Conversely, OTUs that exert large influences in North Sea food webs were not identified as keystone OTUs in co‐occurrence networks. For example, copepods contribute up to 90% of the zooplankton biomass in the North Sea and are responsible for transferring energy from primary producers to commercially important fish species, as well as nutrient recycling and carbon export (Kürten et al. 2013; Mortelmans et al. 2021). We suspect that they fail to have significant correlations with other OTUs as a result of being present in such high abundance across all samples (Zurell et al. 2018). These examples highlight some of the potential flaws associated with both eDNA metabarcoding and correlative relationships, underscoring the need for more robust validation in our interpretation of co‐occurrence networks.

In conclusion, co‐occurrence analyses coupled with eDNA metabarcoding data hold great potential to improve our understanding of the status and functioning of ecosystems, including identifying species interactions and potential bio‐indicator species. Here, co‐occurrence networks and food webs revealed similar trends in ecosystem complexity, despite interactions forming these networks being largely different, making the outcomes challenging to interpret. Trophic interactions formed a small proportion of the established co‐occurrences, leading to key food web components, such as forage fish and copepods not being highly connected in co‐occurrence networks. Therefore, we strongly recommend that co‐occurrence networks should be employed alongside validation methods, such as ground truthing within well‐studied ecosystems, as demonstrated in this study. In this scenario, food webs and co‐occurrence networks are complementary, and co‐occurrence networks highlighted key taxa to focus diet analyses on to overcome current limitations, especially in planktonic communities. Furthermore, limitations of both eDNA metabarcoding and correlation methods need to be explicitly accounted for in these analyses, as both may impact upon which interactions are detected, as demonstrated in the present examination. We encourage future research exploring eDNA metabarcoding studies across different spatio‐temporal scales to optimise the detection of species interactions within co‐occurrence networks. These ecosystem‐level analyses could then provide an early warning system for detecting ecosystem changes in response to climate warming or human pressures and highlight species or mechanisms underpinning the changes, which can contribute to management or policy decisions.

## Author Contributions

E.B., M.B. and S.J.G. conceptualisation. E.B., I.M.C., E.V., M.B. and S.J.G. methodology. E.B. investigation and formal analysis. K.P.R. resources. I.M.C. software. E.B. and I.M.C. data curation. E.B. writing original draft. E.B. visualisation. E.B., K.P.R., E.V., M.B. and S.J.G. writing review. I.M.C., K.P.R., E.V., M.B. and S.J.G. supervision.

## Conflicts of Interest

The authors declare no conflicts of interest.

## Supporting information


Data S1

## Data Availability

The data are archived in the NCBI BioProject repository under accession number PRJNA1069036.
